# Fracture healing in a polytrauma rat model is influenced by mtDNA:cGAS complex mediated pro-inflammation

**DOI:** 10.1186/s40634-023-00637-5

**Published:** 2023-09-01

**Authors:** Preeti J. Muire, Alicia L. Lofgren, Stefanie M. Shiels, Joseph C. Wenke

**Affiliations:** 1 Combat Wound Care, US Army Institute of Surgical Research, JBSA Ft Sam Houston, San Antonio, TX 78234 USA; 2 Department of Orthopaedic Surgery and Rehabilitation, Loyola University Medical Center, Maywood, IL USA; 3 Department of Orthopaedic Surgery and Rehabilitation, University of Texas Medical Branch, Galveston, TX USA; 4 Shriners Children’s Texas, Galveston, TX USA

**Keywords:** Severe trauma, Small molecule drug, Immunomodulator, Bone, DAMPs, Injury

## Abstract

**Purpose:**

The mitochondrial DNA (mtDNA) activated cyclic guanosine monophosphate–adenosine monophosphate synthase—stimulator of interferon genes (cGAS-STING) signaling pathway is a key player in mediating immune responses in autoimmune disorders and cancer. However, its role in severe trauma associated fracture healing is unknown. This study investigated if the cGAS-STING signaling pathway contributes to delayed bone healing in polytrauma (PT) fractures.

**Methods:**

For preliminary analyses, therapeutic dosage of RU.521 (cGAS inhibitor) (*n* = 2) was determined in C57BL/6 J mice by mass spectrometry, and IFNβ expression levels in serum and bronchioalveolar fluid (BALF) at 6 and 24 h (h) in RU.521/vehicle + mtDNA injected mice (*n* = 3/treatment and time point) was measured by ELISA. In the main study, plasma mtDNA was quantified by qPCR in a clinically relevant delayed fracture healing PT rat model with burn injury, blunt trauma, and a femoral fracture at 3 h post-trauma (hpt). Next, PT rats received either RU.521 (12 mg/kg in povidone; *n* = 8) or vehicle (povidone only; *n* = 5) immediately after injury and were followed up for 5 weeks post-trauma to assess bone regeneration by radiography and histology.

**Results:**

IFNβ levels were significantly decreased only at 24 h in BALF of RU.521 treated mice. At 3hpt mtDNA was significantly elevated in PT rats compared to rats without injury. When treated with RU.521, PT rats showed improvement in bone healing compared to vehicle control PT rats.

**Conclusions:**

These data reveal that the cGAS-STING signaling pathway influences trauma-induced delayed bone healing. However, further evaluation of this pathway at the cellular and molecular levels to augment PT associated detrimental effects is needed.

**Supplementary Information:**

The online version contains supplementary material available at 10.1186/s40634-023-00637-5.

## Introduction

Delayed fracture healing is an ongoing problem faced by polytrauma (PT) patients who experience dysregulated immune response [1, 2]. Current research suggests the importance of creating a balance between pro- and anti-inflammatory signaling cascades during early hours following injury to facilitate timely bone healing [1, 3, 4]. The search for effective and promising immunomodulatory therapeutics to mitigate early pro-inflammation and combat delayed fracture healing in PT patients remains active.

Following trauma, the early imbalances of the inflammatory responses are heavily influenced by circulating damage associated molecular patterns (DAMPs) such as mitochondrial DNA (mtDNA), high mobility group box protein 1 (HMGB1), and S100 that leak out from dying cells [5, 6]. While the role of HMGB1 and S100 has been previously studied in PT patients with delayed bone healing scenarios, the role of cell free mtDNA and associated pathways are yet to be explored in the context of PT. In a previous study, we demonstrated that by transient neutralization of HMGB1 and its associated pro-inflammatory pathways we could achieve improved bone fracture healing in a PT rat model [7]. Next, we questioned whether DAMP activated pathways, different from those activated by HMGB1, when modulated can regulate confounding immune responses and maintain proper homeostasis to promote bone healing. To address this, we chose to evaluate the role of the mtDNA-activated pathway i.e., the cyclic guanosine monophosphate–adenosine monophosphate (cGAMP) synthase (cGAS)—stimulator of interferon genes (STING) pathway in our PT fracture model [8].

In pathological conditions extracellular mtDNA from dying cells can gain access to the cytoplasm of neighboring cells and activate the cGAS-STING pathway by binding to cGAS [9, 10]. Circulating mtDNA is abundant in trauma patients with extensive tissue necrosis and is a well-recognized pharmacological target in multi-organ system failure [10]. The activated cGAS:mtDNA complex synthesizes secondary messenger cGAMP to activate stimulator of interferon gene expression protein (STING) to produce the pro-inflammatory cytokine, interferon β (IFNβ), via the translocation of transcription factor, nuclear factor kappa-light-chain-enhancer of activated B cells (NFκB) into the nucleus. Elevated levels of IFNβ leads to metabolic dysfunction and hyper-inflammatory responses involving monocyte and neutrophil expansion causing undesirable outcomes [11].

The cGAS-STING signaling pathway has a role in several inflammatory disorders and studies on myocardial ischemia, traumatic brain injury, and liver damage models suggest that activation of cGAS/STING axis is not only a side effect of the injury, but also can actively contribute to cell death and apoptosis [12]. Other studies have suggested that a prolonged pro-inflammatory state has detrimental effects on fracture healing [13]. To address this issue, we sought to therapeutically block the cGAS pathway with RU.521 immediately following injuries to dampen detrimental pro-inflammatory responses that alter the normal fracture healing process [14]. RU.521 selectively binds to the mtDNA binding site on cGAS, thereby preventing the binding of cell free mtDNA to cGAS and inhibits the activation cGAS:STING pathways [14]. Finally, resulting in inhibition of IFNb expression.

## Material and methods

### Animals and surgical care

In this study, male C57BL/6 J mice (Jackson Laboratory; 12 – 14 weeks; 26 – 34 g) were used to determine the dosing concentration of RU.521 and male Sprague–Dawley rats (Envigo; 10–16 weeks; 354-379 g) were used to evaluate fracture healing. All animals were individually housed in ventilated cages and provided with unlimited access to food and water and unrestricted activity. Research was conducted in compliance with Animal Welfare Act, the implementing Animal Welfare regulations, and the principles of the Guide for the Care and Use of Laboratory Animals. The Institutional Animal Care and Use Committee approved all research conducted in this study. The facility where this research was conducted is fully accredited by the AAALAC International. Animal study protocols (#A-17–027 and #A-20–032) were prepared for all in vivo studies and were approved by the US Army Institute of Surgical Research (USAISR) Institutional Animal Care and Use Committee (IACUC) before starting the study. Prior to surgery or treatment, the animals were randomly assigned to each of the treatment or control groups. This study was carried out in compliance with the ARRIVE guidelines [15].

### mtDNA preparation

Exogenous mtDNA was used to stimulate acute lung injury and systemic inflammation in mice (Lemeng Zhang et al. 2016). MtDNAwas extracted from mouse liver by partly following a previously published protocol [16] with some modifications. Anesthetized mice (*n* = 10) were euthanized via cardiac exsanguination with secondary cervical dislocation and liver collected. Briefly, mitochondria were isolated from homogenized liver samples using a mitochondria isolation kit for tissue (ThermoFisher Scientific) following manufacturer’s instructions. Next, the protein and DNA were isolated by disrupting the mitochondria using five freeze/thaw cycles in liquid nitrogen and water bath at 37 °C. The sample were centrifuged at 12,000xg for 10 min at 4 °C and then 100,000xg for 30 min at 4 °C and the supernatant was further processed for mtDNA extraction and purification using QIAmp DNA blood mini kit (Qiagen) following manufacturer’s recommendations. DNA concentration was determined spectrophotometrically with Nanodrop (Invitrogen) by measuring the absorbance of the sample at 260/280 nm (A_260/280_).

### RU.521 drug preparation

RU.521 (Aobious; Gloucester, MA) was initially solubilized in dimethyl sulfoxide (DMSO) to prepare a 2% drug suspension and further mixed to make a 10% (w/v) DMSO in polyvinylpyrrolidone (povidone) for use. The vehicle control was 10% (w/v) DMSO in povidone. The mice received either 0.614 mg/kg (*n* = 5/time point) or 12 mg/kg (*n* = 2/time point) of RU.521 subcutaneously (SQ). Forty-five minutes and two hours (h) following dosing, animals were anesthetized, blood was collected in EDTA coated tubes and the mice were euthanized via cardiac exsanguination, with a secondary euthanasia by cervical dislocation. Blood was centrifuged at 1000xg for 10 min and plasma was collected to measure RU.521 concentration via mass spectrometry. In mice that received a low dose of RU.521 i.e., 0.614 mg/kg, the plasma drug concentrations did not reach the desired target concentration at both 45 min and 2 h post dosing. However, in mice that received a high dose of RU.521 i.e., 12 mg/kg, the plasma drug concentration was detected to be greater than 1 µM at 45 min post injection and the drug concentration dropped below 1 µM after 2 h post dosing (Supplementary Fig. 1). Based on this evaluation, we decided to use 12 mg/kg RU.521 for the rest of the study.

### Mass spectrometry

A target concentration of 1 µM for RU.521 used in this study was determined based on an in vitro experiment (IC75 of 1 µM) as previously published [14]. RU.521 in 100 µl of plasma samples was extracted with 4 volumes ethyl acetate followed by centrifugation at 1000xg for 10 min. The organic fraction was separated and dried under ultra-high purity nitrogen at room temperature. Dried samples were resuspended in 100µL acetonitrile-formic acid (99.9:0.1, v/v) and analyzed by UPLC-MS/MS using a Waters Acquity H-class ultra-performance liquid chromatography system (Waters, USA) coupled to a Waters Xevo G2-XS quadrupole time of flight mass spectrometer (Waters,USA) equipped with an electrospray ionization (ESI) source. RU.521 was resolved on a Phenomenex Kinetex C18 column (1.7 µm, 2.1 × 50 mm) using water-formic acid (99.9:0.1,v/v) as mobile phase A and acetonitrile-formic acid (99.9:0.1,v/v) as mobile phase B. Isocratic elution was performed at 20:80 A:B for 5 min at a flow rate of 0.4 mL/min. The column temperature was set to 40 °C. Three microliters of resuspended sampled was injected on the column with the auto-sampler held at 8 °C. Single reaction monitoring (SRM) for RU.521 was performed in positive ion mode at m/z 415.035 → 385.058. Optimized values for collision energy (CE), cone voltage, capillary voltage, source temperature and desolvation gas flow rate were 25 (nominal), 0 (nominal), 3 kV, 80 °C, and 600L/hr, respectively. RU.521 concentration in samples was interpolated from the standard curve using least-squares linear regression curve fitting with 1/x^2^ weighting.

### Effectivity of RU.521 in the presence of exogenous mtDNA

Mice either received 12 m mg/kg RU.521 (*n *= 6 or similar volume of DMSO/povidone (vehicle) (*n* = 6) SQ. One hour later, all mice were anesthetized and intraperitoneally injected with 1.3 mg/kg of mtDNA. The mtDNA concentration was selected from a similar published study [16]. Mice were recovered in clean cages with continued monitoring. Six (*n* = 3 for RU.521 and *n* = 3 for vehicle) and twenty-four (*n* = 3 for RU.521 and *n* = 3 for vehicle) hours post mtDNA injections, mice were anesthetized, blood was collected intracardiac (IC) and animals were euthanized via cardiac exsanguination with secondary euthanasia via cervical dislocation. Lungs were harvested, infused with 100 µl of sterile PBS via the trachea and the bronchoalveolar lavage fluid (BALF) was collected. In the same manner and on the same day blood and BALF was also collected from naive animals (*n* = 2) that did not receive any mtDNA or RU.521 injections. All samples were immediately centrifuged at 1000xg for 10 min and the serum and BALF were collected and flash frozen in liquid nitrogen. Serum and BALF samples were stored at 80 °C until assayed.

### Enzyme Linked Immunosorbent Assay (ELISA)

Serum and BALF IFNβ concentrations were determined using Legend max™ mouse IFNβ ELISA kit (BioLegend) following manufacturer’s instructions.

### Polytraumatic fracture model: surgery and RU.521 treatment

A previously established rat PT model with delayed fracture healing was used in this study to evaluate bone healing [17]. Prior to surgery, all animals received preemptive buprenorphine SR-LAB (1.2 mg/kg, SQ) at least 15 min before surgery. All rats were anesthetized and maintained with 1–3% isoflurane in 90–95% oxygen from a concentrator delivered via a nose cone on a Bain circuit connected to the rodent gas anesthesia machine (VetEquip Inc., Pleasanton, CA). Briefly, under general anesthesia, a polyacetyl plate was affixed to the femur prior to removal of a 3 mm bone segment by reciprocating saw under copious saline irrigation. Prior to creating the osteotomy, the periosteum was removed by scrapping it off the bone. Tissues were approximated and closed with 3–0 vicryl suture and skin clips. Following closure and confirmatory radiographs (UltraFocus 100, Faxitron Bioptics, Tucson, AZ), the animal was immediately place supine in a controlled drop-weight device where a 0.3 kg weight was dropped from 68 cm to exert 2 J of energy on the rat’s thorax. Following assurance of the animal’s breathing and level of anesthesia, the animals were placed supine in a custom mold and shaved dorsal skin submerged in 100 °C water for 10 s. Immediately following trauma procedures, rats received RU.521 (*n* = 13, 12 mg/kg) or vehicle (*n* = 7) SQ. Plain radiographs were immediately collected prior to recovery and at three weeks using a Faxitron Ultrafocus. Rats were closely monitored for 72 h and then at least once weekly for signs of pain and distress, such as changes in behavior, appetite, and body weight. Rats with ≥ 10% body weight loss relative to pre-surgery weight received 3 ml normal sterile saline SQ daily until able to maintain weight. At five weeks following trauma (wpt), anesthetized rats had blood collected IC and were humanely euthanized by cardiac exsanguination with secondary euthanasia via thoracotomy. The fractured femurs were harvested and ex-vivoradiographs were taken, and then fixed in formalin for histology. Following surgery and recovery, one rat from the RU.521 group was found dead in cage on day 9 from apparent failure to thrive and one rat from the vehicle group was euthanized by cardiac exsanguination with secondary euthanasia via thoracotomy on day 4 due to improper placement of the burn, which caused the animal to chew. Three more rats from RU.521 block did not survive (one had excessive bleeding during segmental defect creation and was found dead 5 h post-traumas, two were found dead in cage from apparent failure to thrive at 48 h assessment) and one more rat from the vehicle group was euthanized by cardiac exsanguination with secondary euthanasia via thoracotomy at 96 h due to failure to thrive. Additionally, one rat from the RU.521 group that demonstrated consistent weight loss over an extended period and needed extra dietary enrichment and multiple saline injections compared to the other rats in the same block was excluded from the study. Because of the mortalities in the RU.521 (33.3%) and vehicle (28.6%) groups, the final number of animals in each group was *n* = 8 and *n* = 5, respectively (Supplementary table 1).

### DNA isolation and RT-qPCR

The mtDNA copy number was determined in plasma from uninjured/naive rats, rats with a single injury and PT rats. Briefly, in a separate cohort, blood was collected IC from anesthetized uninjured rats (naive) (*n* = 3), fracture only/single injury (*n* = 3) and from PT rats (*n* = 3) at 3 h following trauma, were humanely euthanized by cardiac exsanguination with secondary thoracotomy. Blood was collected in EDTA tubes and centrifuged at 1000xg for 10 min. Plasma was collected and total DNA was isolated from the plasma using QIAmp DNA blood isolation kit (Qiagen) according to the manufacturer’s protocol. Total DNA was quantified spectrophotometrically using a Nanodrop 2000 Spectrometry (Thermo Scientific). DNA isolated from plasma was used to assess mtDNA copy number by RT-qPCR using the mtDNA copy number kit (MCN2) from Detroit R&D, Inc (Detroit, MI) according to the manufacturer’s protocol. MtDNA copy number was determined using the delta-delta CT method.

### Micro-computed tomography (μCT)

The µCT (SkyScan 1276, Bruker, Kontich, Belgium) was used to assess bone formation within the fracture defect. Each defect was scanned at a resolution of 13 μm voxel size at 70 kV, 200 μA, with frame averaging of 2, in 360° using the Al 0.5 mm filter. Images were reconstructed using NRecon (Bruker, Kontich, Belgium) with the attenuation coefficient set to 0–0.06 and manual post-alignment correction. Image stacks were reoriented with DataViewer (Bruker, Kontich, Belgium) so that the transaxial slices were in the plane of the defect border. Three-dimensional analyses were completed using CTan (Bruker, Kontich, Belgium). The volume of interest (VOI) within the defect extends to the last slice proximally and distally in which no cortical bone is present. The bone volume and bone volume fraction (BV/TV) of the mineralized tissue were calculated within the region of interest (ROI) drawn to cover the defect site (i.e. average length of 3.07 ± 0.09 mm) for each sample. The defect ROI used a polygonal shape around the cortex at the slice in which an intact cortex was first seen, both proximal and distal to the defect, and interpolated throughout the VOI to measure new bone growth within the void created by the excised femur. A new ROI was drawn at least every 25 slices, based on the rapidity of change in callus shape, and interpolated between. A global threshold of 92 was calculated by averaging the value given for each sample using the auto-threshold algorithm in CTan, which uses the Otsu method [18]. The reported method of global thresholding was evaluated alongside Otsu calculations used previously and was found to be comparable. Using the same Defect ROI and the same threshold across all samples, a two-dimensional analysis was then performed to distinguish mineralized tissue from non-mineralized tissue within the defect. The cross-sectional bone area (mean ± SEM) was calculated for each transaxial slice, converted to a percent to allow for differing defect lengths between animals, and plotted across the defect extending from the proximal (0%) to the distal (100%) edge. Values are shown as bone area (mm^2). Scorers were not blind to the treatment, instead, the scoring was completely determined by the program.

### Histology

Formalin fixed femurs were decalcified in formic acid for 21 days and embedded in paraffin. Longitudinal sections of 8 μm thickness were stained with hematoxylin and eosin (H&E) for descriptive assessment of tissue morphology. Images were captured on an Olympus BX41 microscope using CellSens standard software (Olympus). At the time of scoring analysis, the scorer was blinded to treatment, but investigator was not blinded to treatment labels post-analysis.

### Statistical analysis and sample size calculation

Statistical analysis was performed using GraphPad Prism version 8.0.0 for Windows (San Diego, California USA). All data were assessed for normality using the Q-Q plot, homoscedasticity plot, residual plot and D'Agostino and Pearson test. The IFNβ protein data were assessed using one-tailed Student’s T test. MtDNA copy number data were assessed using one-tailed Student’s T test with Mann–Whitney test to test for differences. For the bone healing experiment, rats were used in two blocks with *n* = 5 (RU.521) or *n* = 5 (vehicle) groups in the first block. The results from the first block of animals indicted that the RU.521 treated rats had > 25% increase in bone growth compared to the vehicle control rats. Average BV/TV% ± standard deviation data computed from the first block i.e., RU.521 treatment = 10.97 ± 7.88 and vehicle = 5.14 ± 1.08 was used to calculate the sample size for the remaining study. Based on the sample size (after excluding post-surgery mortalities) of *n* = 4 (RU.521) and *n* = 4 (vehicle), an alpha of 0.05, and assuming unequal variance there was a 43% power to observe statistical significance between the RU.521 treatment and vehicle using a two-sample, one-sided t-test. A sample size of additional *n* = 8 (RU.521) and *n* = 2 (vehicle) was calculated for the second block with the assumption that the additional animals will give us 81% power to observe statistical differences between the groups (i.e., alpha of 0.05). MicroCT data evaluating bone regeneration within the segmental defect of RU.521 or vehicle groups were assessed by one-tailed Student’s T test with Welch’s correction for unequal sample size. Bone mineralization data were assessed by two-tailed Student’s T test with Welch’s correction for unequal sample size. A *p*-value < 0.05 was deemed significant. All results reported as mean values and the error bar represent standard error mean (SEM).

## Results

### RU.521 effect on inflammation

**Fig. 1:**
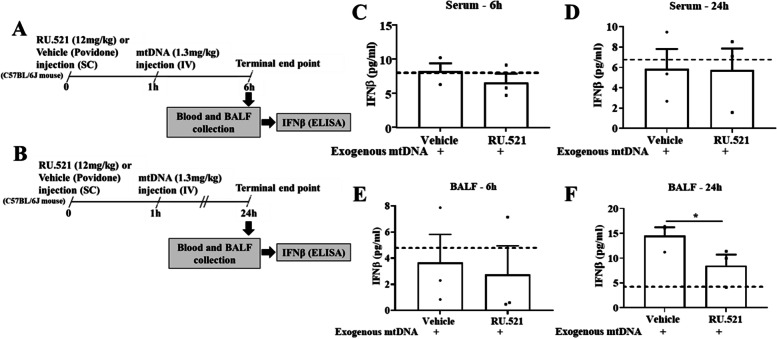
(**A**) Levels of IFNβ in broncho alveolar fluid (BALF) at 6 h; (**B**) Levels of IFNβ in BALF at 24 h.; (**C**) Serum IFNβ at 6 h; Levels of IFNβ in BALF at 6 h; (**D**) Serum IFNβ at 24 h; Levels of IFNβ in BALF at 24 h. Dotted line represents data from naïve animals (*n* = 2) that did not receive any mtDNA or RU.521 injections. *p ≤ 0.05 comparing IFNβ protein levels in vehicle (*n* = 3/time point) and RU.521 (*n* = 3/time point) treated mice. Data are graphically represented as mean ± SEM with each animal represented as a single point

Blood and BALF were collected from mtDNA exposed mice at 6 h and 24 h post RU.521 or vehicle treatment (Figs. 1A and B). In RU.521 treated mtDNA exposed mice there was no difference in IFNβ levels at 6 h in bronchiolar alveolar lavage fluid (BALF) (Fig. 1C), but statistically significant decrease in IFNβ levels in the BALF at 24 h (Fig. 1D). Further, there were no differences in IFNβ levels in the serum at either endpoint (Figs. 1E and F).

### mtDNA copy number

**Fig. 2:**
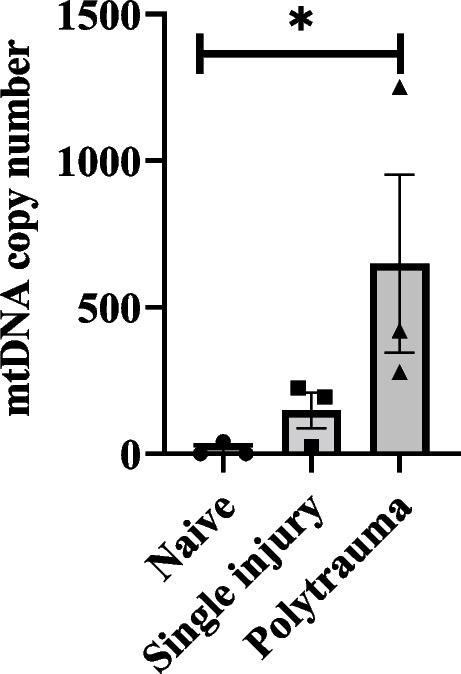
Mitochondrial DNA (mtDNA) copy numbers in plasma from uninjured (naïve) rats (*n* = 3), single injury rats with osteotomy (*n* = 3) and polytrauma (PT) rats (*n* = 3) at 3 h post-trauma (hpt). *p = 0.05 comparing PT to naïve and single injury rats. Data are graphically represented as mean ± SEM with each animal represented as a single point

The circulating mtDNA copy numbers were significantly higher in the PT rats compared to the naïve rats (*p* = 0.05) (Fig. 2).

### Fracture healing in PT rats following treatment with RU.521

**Fig. 3:**
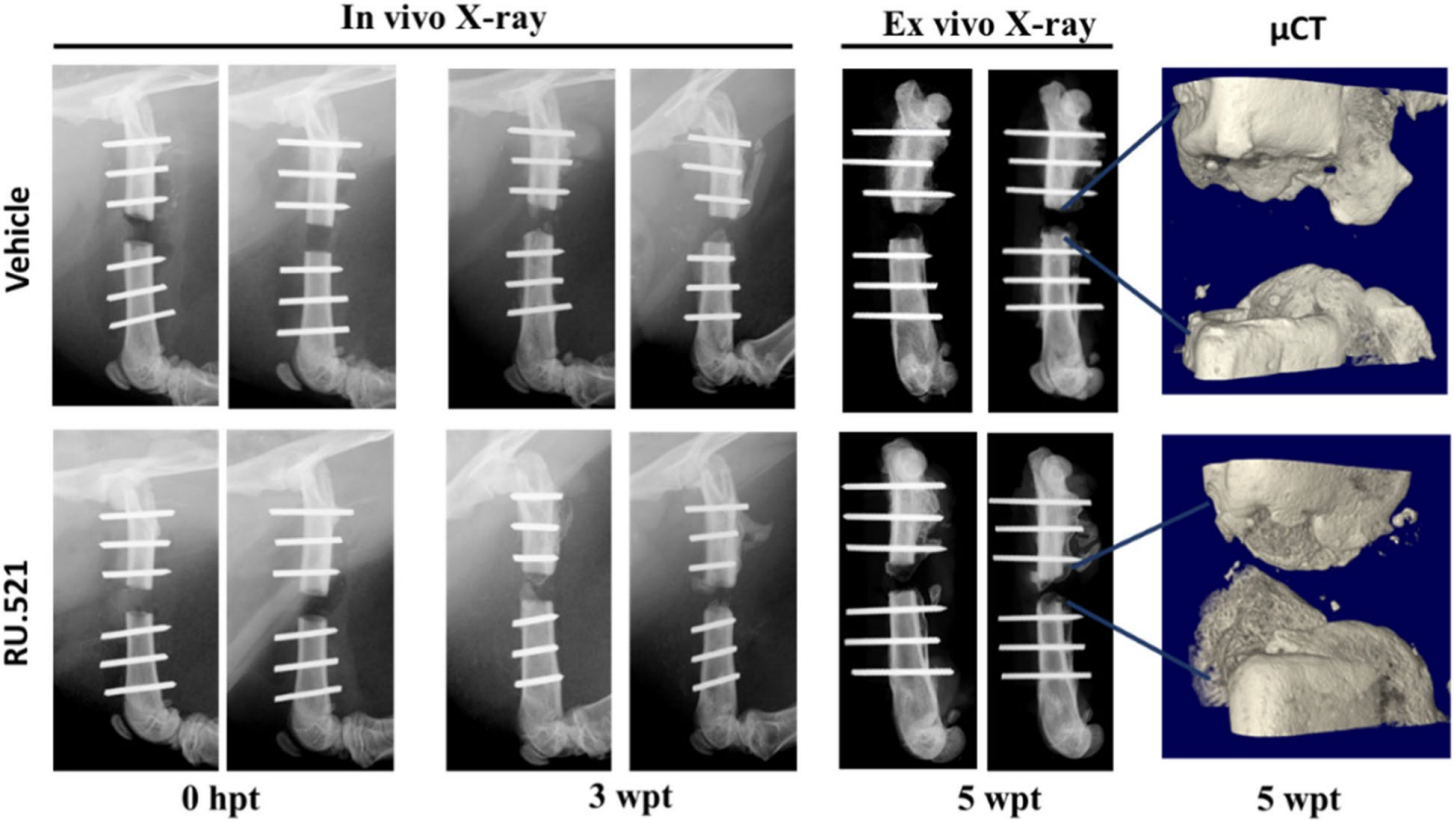
Radiographic and micro-computed tomography images of the fractured callus at 0 h post-trauma (hpt), 3 weeks post-trauma (wpt) and 5 wpt. Two representative images of vehicle (*n* = 5) and RU.521 (*n* = 8) are shown

**Fig. 4:**
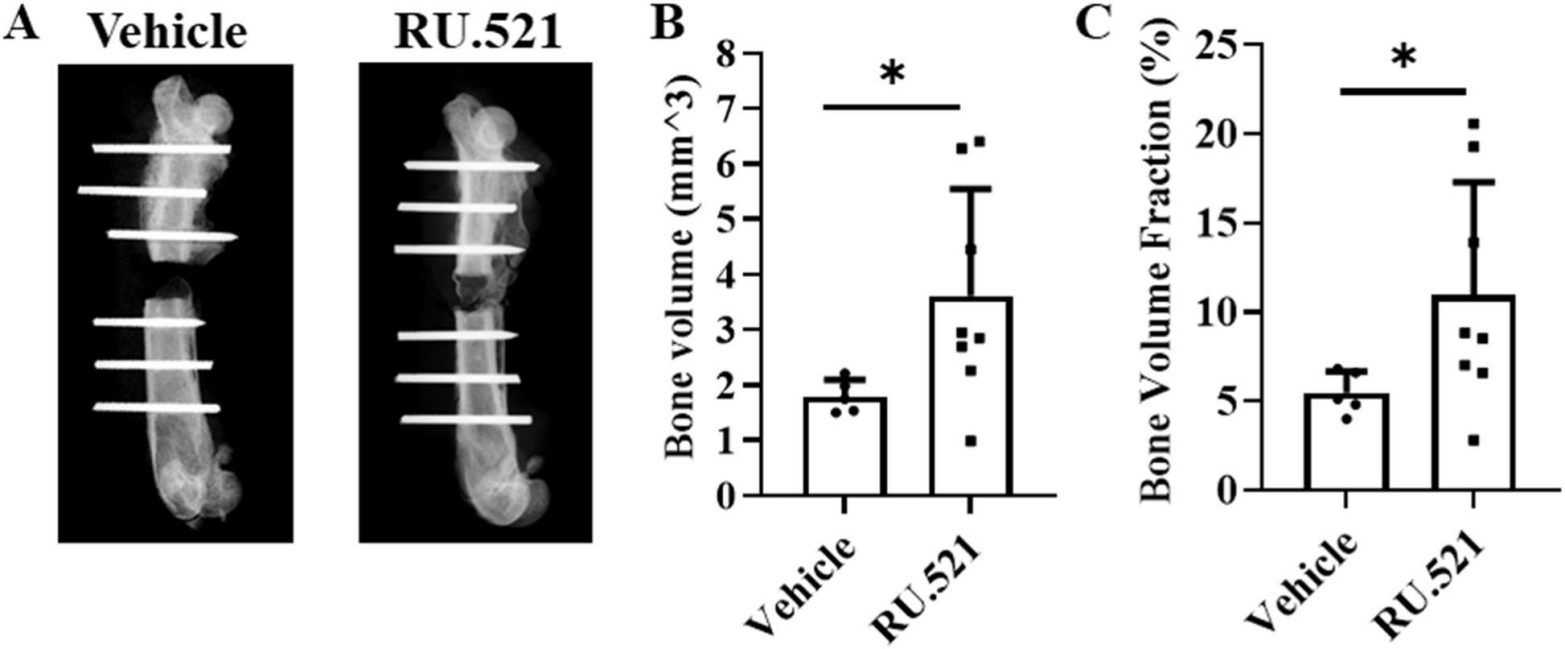
Micro-computed tomography (μCT) analysis at 5 weeks post trauma indicates that RU.521 treatment improves bone formation after fracture in PT rats. (**A**) Representation of the VOI analyzed depicting the space in which the excised bone used to be, ‘Defect’ (**B**) Bone Volume (mm^3) results from the Defect VOI; (**C**) Bone Volume Fraction (%) results from the Defect VOI. (*n* = 5/vehicle control and *n* = 8/RU.521 treatment). * *p* < 0.05 vehicle control PT rats vs. RU.521 treated PT rats. Data are graphically represented as mean ± SD

The representative images obtained from control and treated PT rats by X-ray imaging performed at 0, 3, and 5 weeks post trauma (wpt) and µCT scans at 5wpt are presented in Fig. 3. At 5wpt, there was a significant increase in bone growth at the fracture site in treated PT rats compared to the control rats at 5wpt (*p* = 0.03 and *p* = 0.04, respectively) (Figs. 4A - C).

**Fig. 5:**
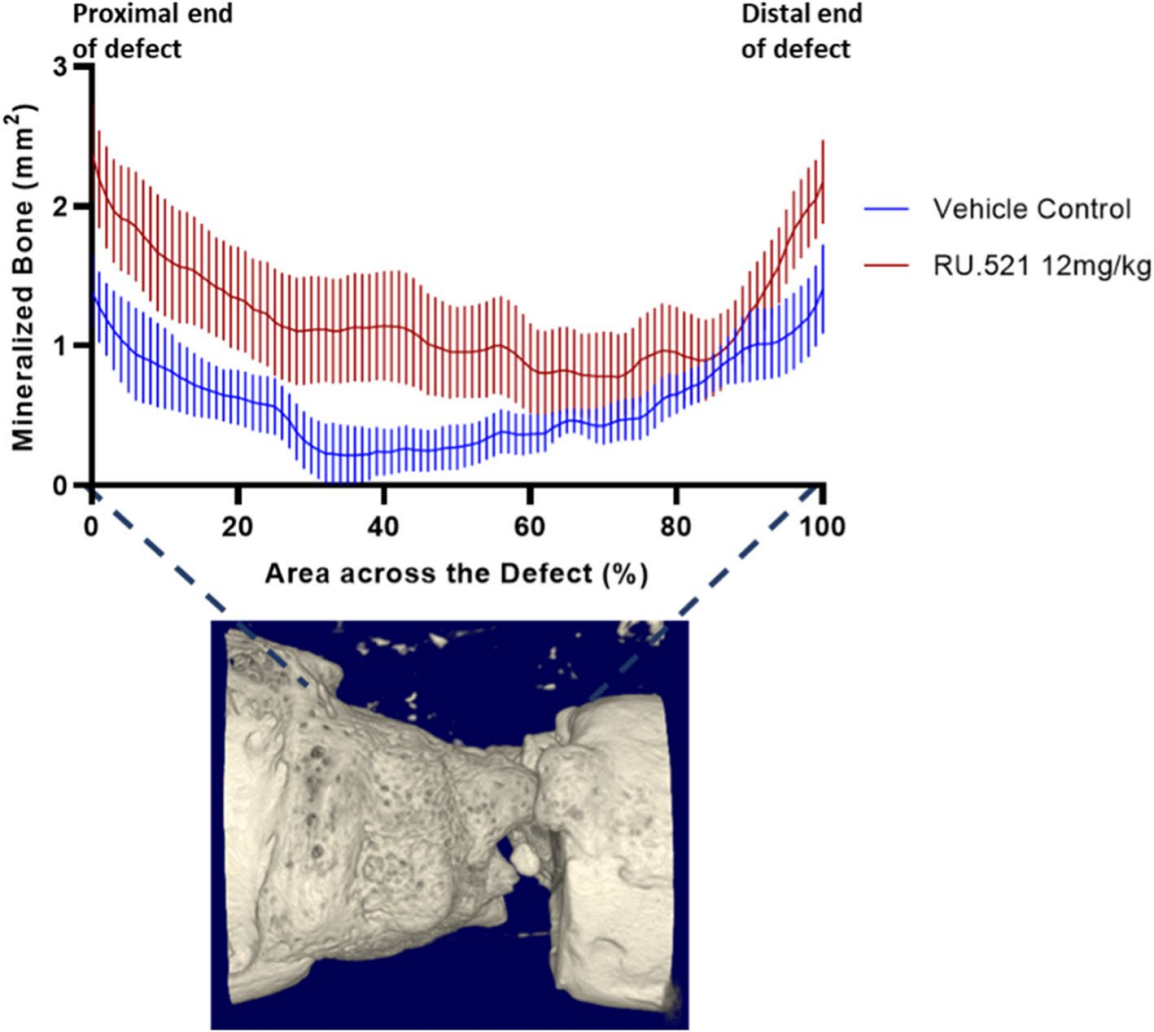
Quantitative differences in the spatial patterns of bone regeneration after five weeks post trauma (wpt). The figure shows the bone area across the 3.07 ± 0.09 mm distance, represented as percent of defect, extending from the proximal (0%) to the distal (100%) defect borders. The cross-sectional bone area (mean ± SEM) was calculated for each trans axial slice (13 µm) to access spatial bone regeneration and remodeling trends for vehicle and RU.521 treatment groups after 5 weeks post treatment. *n* = 5/vehicle control and *n* = 8/RU.521 treatment. Data are graphically represented as mean ± SEM

Quantitative differences in the spatial patterns of bone growth following 5wpt in control and RU.521 treated PT rats show the regenerated bone distribution within an average of 3.07 ± 0.09 mm distance, represented as percent of defect, extending from the proximal (0%) to the distal (100%) defect borders for both groups. The bandwidth corresponds to variations in bone regeneration in each 13 µm thickness of mineralized tissue slice between different samples within a group. Statistical evaluation of the mineralized tissue slice at 50% within the area across the defect confirms significant mineralized bone formation in the RU.521 treated PT rats (*p* < 0.45) (Fig. 5).

### Histological evaluation

**Fig. 6:**
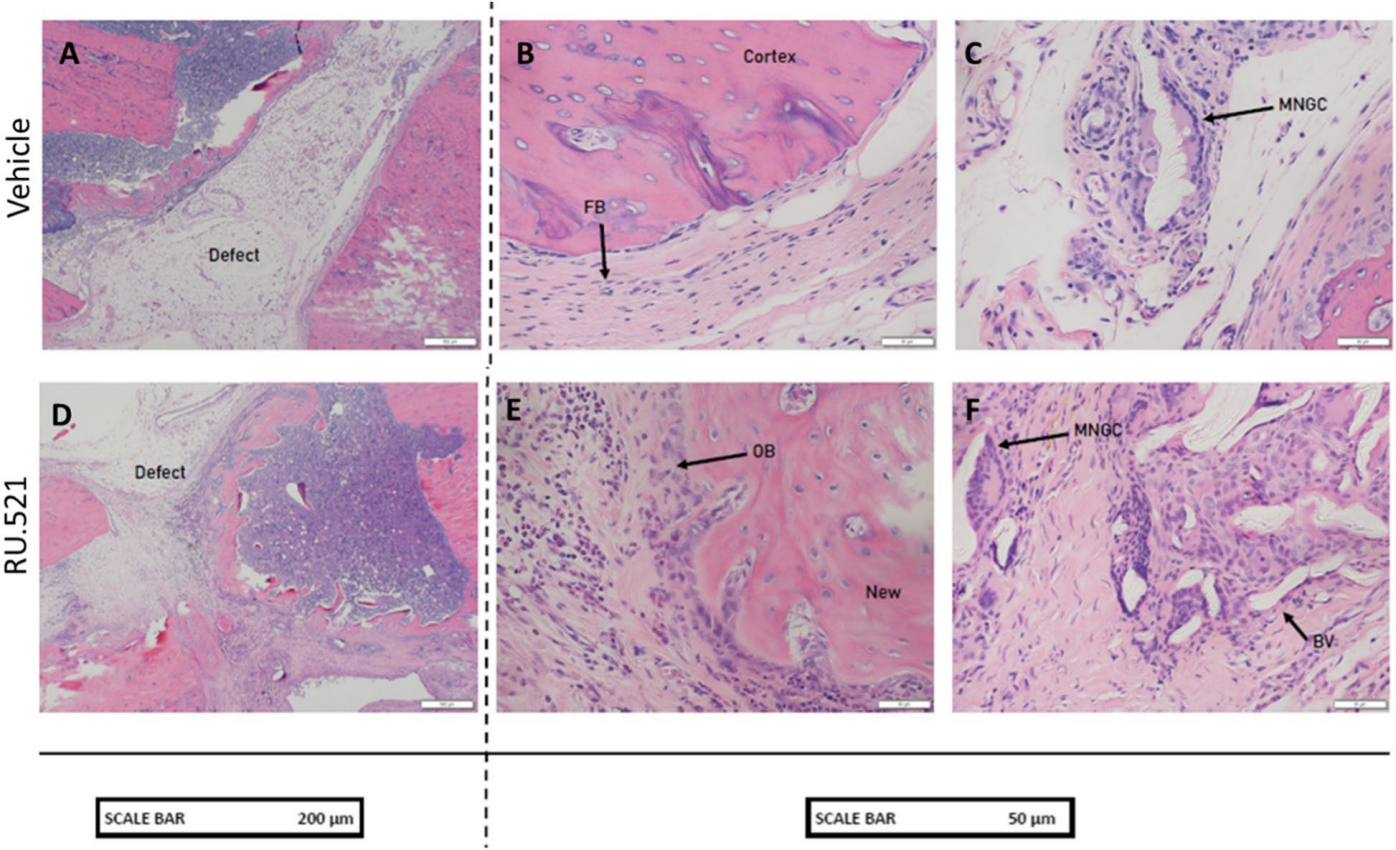
Histological analysis of fractured bones of polytrauma (PT) rats that received vehicle or RU.521. Paraffin embedded bone samples were stained with hematoxylin and eosin (H&E) stain. MNGC – multinucleated giant cells; OB – osteoblasts; FB – fibroblasts; BV – blood vessel; New – new bone formation; Cortex – existing cortical bone; and Defect – defect created during surgery

In a bone section representing the fracture site of control PT rats at 5wpt, the defect was mostly filled with adipose tissue and a scattering of new blood vessels. There were regions of empty lacunae, a sign of necrotic bone, within ~ 300 µm of the cut ends of the defect. These regions were either surrounded by layers of fibrous tissue, including fibroblasts, or a thin layer of new bone formation surrounded by osteoblasts. Most of the new callus formation stemmed from the outer cortical region of the diaphysis (the periosteum was removed during surgery). A few multinucleated giant cells were identified in the marrow and near, but not touching, the new callus formation (Fig. 6 A – C). In a section of bone from the RU.521 treated rats, the defect is mostly filled with boney callus and fibrous connective tissue. Boney callus extends both from the proximal cut end into the defect space and from the outer cortex. There are chondrocytes within the callus, indicative of endochondral bone formation. As with the other group, the cut ends are riddled with empty lacunae, a traditional indication of necrotic/unhealthy tissue (probably from the sawing). The callus is surrounded by both thick layers of osteoblasts and numerous fibroblasts. There are numerous multinucleated giant cells near but not touching callus formation outside the defect region (Fig. 6 D – F).

## Discussion

**Fig. 7:**
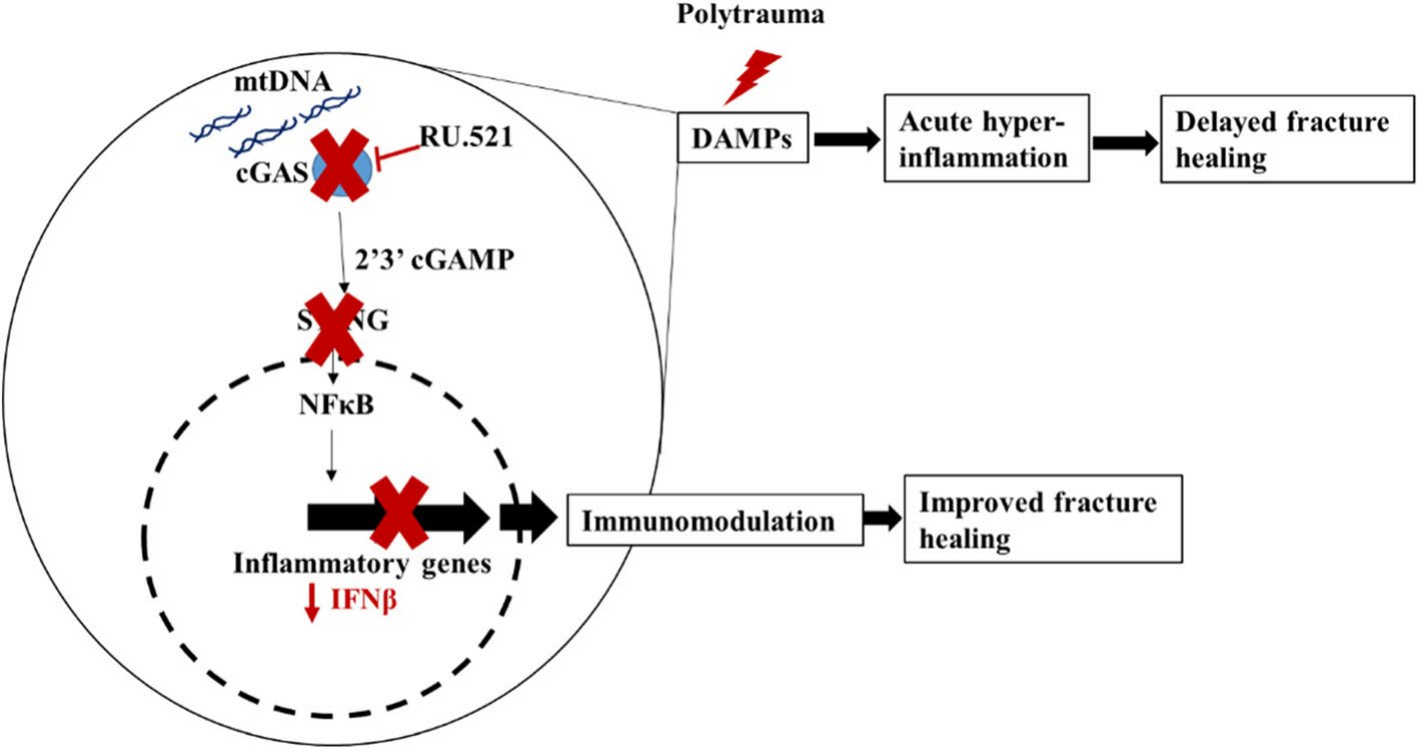
Graphical illustration of the mechanism underlying the immunomodulation driven improved fracture healing mediated by RU.521 inhibition of the cyclic guanosine monophosphate–adenosine monophosphate (cGAMP) synthase (cGAS)—stimulator of interferon genes (STING) signaling pathway in a polytrauma (PT)

This study followed a three-step approach to assess the role of cGAS in PT fracture healing. In the first step, the dosing strategy for RU.521 was determined. The rapid decline of the circulating RU.521 concentration from ~ 1.2 nM at 45 min to 0.7 nM at 2 h post injections could suggest its short half-life or that it is engaging with its target cGAS intracellularly and blocking the pathway. Next, serum IFNβ levels were evaluated in mtDNA exposed mice to confirm cGAS pathway inhibition following treatment with RU.521. Decreased levels of IFNβ expression in the BALF at 24 h following RU.521 treatment indicates the effectiveness of RU.521 in inhibiting cGAS pathway in vivo at 12 mg/kg. Notably, the lack of difference in serum IFNβ expression at 24 h indicated that the activity of the drug might be tissue specific, especially because extracellular mtDNA induces acute lung injury [16]. These results about IFNβ expression were supported by another study in which THP1 cells (human macrophage cell line) were stimulated with PAMPs and treated with RU.521, where it was shown that the RU.521 at EC50 of 0.70 µM inhibited IFNβ and IL6 expression in vitro [14]. Lastly, after confirming elevated levels of circulating mtDNA in a clinically relevant PT rat model with delayed fracture healing, we tested the hypothesis that inhibition of the mtDNA-cGAS inflammatory pathway at the point of injury would improve bone healing in a PT fracture model (Fig. 7). The data from our experiment confirmed this hypothesis. We demonstrated that blocking cGAS increased osseous formation at the fracture site compared to the vehicle control. The increased boney callus and the presence of osteoblasts surrounding the callus region further supported our hypothesis. These findings, although preliminary, are novel in the field and they strongly indicate the activation of mtDNA-cGAS inflammatory pathway during the early hours post polytraumatic injuries. Thereby supporting the consensus that dysregulated inflammation occurs during those early time points, cGAS might be among the several other essential modulators of the early immune response in the severely injured.

Currently used therapies for non-healing fractures, such as bone morphogenetic proteins, parathyroid hormone and bone graft substitutes have been promising in the clinics but only to a limited capacity especially in the case of PT where inflammation significantly influences fracture healing [13, 19]. In such cases, the application of immunomodulatory drugs and biologics that attenuate deranged inflammation may be advantageous when applied in synergy with traditionally used approaches to promote bone healing. Additionally, several immunomodulatory drugs are stable, non-immunogenic, highly soluble, faster, safer, and cheaper making them highly desirable. Considering the role of cGAS in altering inflammatory responses in various pathologies, it is most likely to contribute to delayed bone healing in PT. Four out of eight PT rats treated with RU.521 demonstrated increased bony formation and the remaining had partial bone formation within the defect. Whereas bony formation was significantly less in all vehicle control PT rats compared to treated PT rats. In chronic pro-inflammatory state TNFα and NFκB signaling pathways are continuously upregulated, leading to further bone breakdown by increasing the number of osteoclasts and decreased osteoblasts [20, 21]. The presence of osteoblasts surrounding the callus region of the RU.521 treated rats further indicates bone regeneration. Not too dissimilar, a study using RU.521 to inhibit the cGAS pathway demonstrated improved cardiac function in mice with sepsis, where treatment with RU.521 alleviated the inflammatory response and prevented further cardiac damage induced by oxidative stress [12]. Similar outcomes have been recently observed in a mouse fracture model where the inhibition of STING demonstrated improved bone healing compared to untreated control suggesting the importance of the inflammatory pathway in bone healing [22]. Among the immune cells, the macrophages are believed to be heavily dependent on the cGAS pathway for detecting and signaling their targets. They are transformed to the reparative phenotype following inhibition of cGAS thereby promoting repair [23].

This is the first study that has assessed the role of cGAS in PT fracture healing. We found that blocking the cGAS pathway increased mineralization in the callus. While these results provide an understanding of the fundamental importance of the cGAS pathway in bone healing, we acknowledge that this is a preliminary study, and further evaluation of the underlying mechanisms is needed. Apart from that, this study has its limitations. The bone healing results had statistical significance in favor of improved bone healing in RU.521 treated PT rats, but not to the point that RU.521 would clinically improve fracture healing, thereby lacking clinically meaningful bone formation. A possible reason for lack of complete bone bridging despite the inhibition of cGAS pathway, is the activation of other DAMP activated pro-inflammatory pathways that influence the bone healing process. We chose 5wpt as the endpoint in this study to evaluate bone healing similar to other fracture healing studies [24]. Still information on later time points is necessary to provide us if complete closure of the fracture site is achievable with the applied treatment regimen. The expression dynamics of the inflammatory cytokines was not evaluated in this study because we focused on the bone healing outcomes. However, inflammation and immune status will need to be addressed in the future studies. This study utilized two different rodent species for the following reasons: (1) due to the lack of availability of molecular tools to determine protein expression of serum IFNβ in rats we performed our initial evaluation in a mtDNA exposed mice and (2) due to technical and surgical difficulties accompanied by increased mortalities during creation of the mouse PT model with delayed fracture healing we proceeded to evaluate bone healing outcome using the RU.521 dosage that was determined in mice in a pre-established PT rat model. Lastly, this study assessed the transient effects of cGAS inhibition on delayed bone healing in PT rats that received a single dose, but a multiple-dosing regimen was not performed. Future studies can check the outcomes following multiple dosing time points to confirm the efficacy of the treatments and their effects on long-term bone healing. Nevertheless, the purpose of the selected time for dosing is the highlight of the study because of the acute hyper-inflammatory response occurring during the initial hours post-injury prompting the use of interventions closest to the most likely point of injury [25].

## Conclusion

The present study demonstrated that inhibition of cGAS with RU.521 treatment improved bone healing compared to the vehicle treatment in PT rats with fractures. The findings also imply that, RU.521 is a potentially beneficial therapeutic agent for immune modulation and bone healing in PT at the recommended dose. While the observations from this study shed light on the potential role of the cGAS pathway in PT patients, further investigation is needed to confirm the therapeutic role of blocking cGAS in humans. Finally, more studies are needed to elucidate if modulating more than one of the DAMP activated pathways would promote complete bone bridging.

### Supplementary Information

**Additional file 1: Supplementary Figure 1.** Circulating RU.521 when administered at a concentration of 0.614mg/kg (*n*=4/time point) and 12mg/kg (*n*=2/time point) in mouse plasma at 45minutes and 2 hours with a target concentration of 1µM.

**Additional file 2: Supplementary Table 1. **The table presents the number of rats used in the study and percentages of mortalities within each group. Group 1 represents rats prior to power analysis and group 2 represents rats after power analysis based on the data derived from group 1 rats.

## Data Availability

All data generated or analyzed during this study are included in this published article.
