# Supplementary figures and images for: Fracture healing in a polytrauma rat model is influenced by mtDNA:cGAS complex mediated pro-inflammation
Source: J Exp Orthop. 2023 Sep 1;10:90. doi: 10.1186/s40634-023-00637-5 (PMC10473996; doi:10.1186/s40634-023-00637-5)

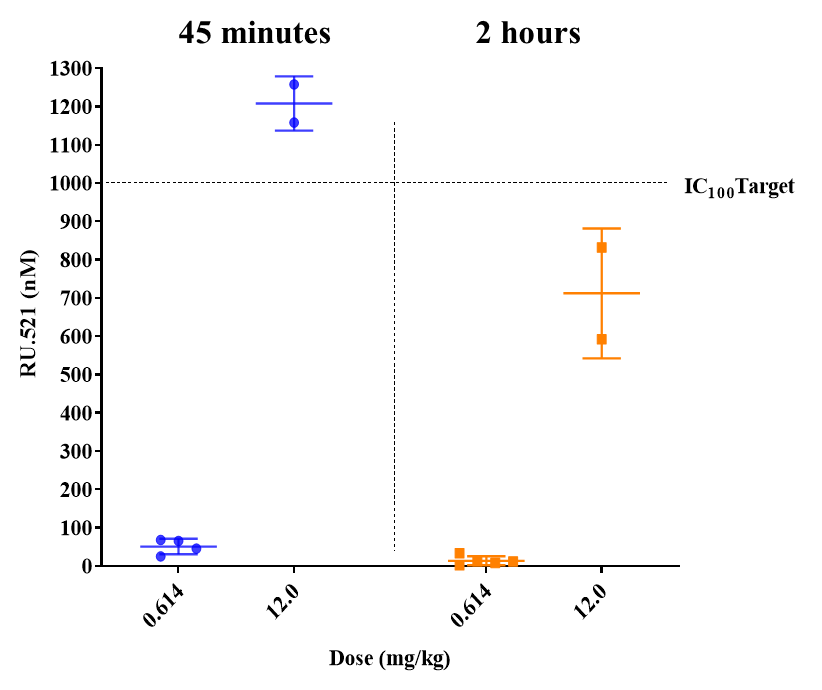

Supplement: Supplementary file 1 — Additional file 1: Supplementary Figure 1. Circulating RU.521 when administered at a concentration of 0.614mg/kg (n=4/time point) and 12mg/kg (n=2/time point) in mouse plasma at 45minutes and 2 hours with a target concentration of 1µM. [file 40634_2023_637_MOESM1_ESM.docx]
