# Supplementary material for: Fracture healing in a polytrauma rat model is influenced by mtDNA:cGAS complex mediated pro-inflammation
Source: J Exp Orthop. 2023 Sep 1;10:90. doi: 10.1186/s40634-023-00637-5 (PMC10473996; doi:10.1186/s40634-023-00637-5)
Supplement: Supplementary file 2 — Additional file 2: Supplementary Table 1. The table presents the number of rats used in the study and percentages of mortalities within each group. Group 1 represents rats prior to power analysis and group 2 represents rats after power analysis based on the data derived from group 1 rats. [file 40634_2023_637_MOESM2_ESM.pdf]

	Vehicle	RU.521
Group 1 (n)	5	5
Survived	4	4
Died	1	1
Group 2 (n)	2	7
Survived	1	5
Died	1	3
Total (n)	7	12
Survived	5	8
Died	2	4
**Group 1 %**	**20.0**	**20.0**
**Group 2 %**	**50.0**	**42.9**
**Total %**	**28.6**	**33.3**
